# Early hyperbaric oxygen effects on neuropathic pain and nitric oxide synthase isoforms in CCI rats

**DOI:** 10.18632/oncotarget.23867

**Published:** 2018-01-03

**Authors:** Yuanyuan Ding, Peng Yao, Tao Hong, Zhenkai Han, Baisong Zhao, Weimin Chen, Guangyu Zhou

**Affiliations:** ^1^ Department of Pain Management, Shengjing Hospital of China Medical University, Shenyang, China; ^2^ Department of Anesthesiology, Shengjing Hospital of China Medical University, Shenyang, China; ^3^ Department of Anesthesiology, Guangzhou Women and Children’s Medical Center, Guangzhou, China; ^4^ Department of Nephrology, Shengjing Hospital of China Medical University, Shenyang, China

**Keywords:** HBO, pain-related behaviors, NOS, neuropathic pain, chronic constriction injury

## Abstract

Neuropathic pain is pain caused by injury or dysfunction in the central and/or peripheral nervous system. Neuropathic pain has a high incidence with a complex mechanism, but effective treatment remains elusive. Hyperbaric oxygen (HBO) therapy has been widely used in the treatment of a variety of neurological diseases. The current study used a rat model of neuropathic pain induced by chronic constriction injury (CCI) of the sciatic nerve. We observed the effects of early use of 2.5 absolute atmosphere (ATA) HBO on neuropathic pain-related behaviors and the expression of nitric oxide synthase (NOS) isoforms in the spinal dorsal horn. In the CCI group, mechanical withdrawal threshold (MWT) was decreased, Thermal withdrawal latency (TWL) was shortened, and mRNA and protein levels of iNOS and nNOS were significantly increased compared to the sham group. MWT was increased, TWL was enhanced, and iNOS and nNOS levels were significantly decreased in the HBO group compared to the CCI group. There was no change in eNOS levels across all groups. HBO treatment at early stages can improve hyperalgesia.

## INTRODUCTION

Neuropathic pain (NP) [1] is pain caused by injury or dysfunction in the central and/or peripheral nervous system, which usually presents with allodynia, hyperalgesia, dysesthesia, and both central and peripheral sensitization as its main clinical features [2]. NP has a high incidence [3] with a complex mechanism; however, effective treatment remains elusive. Therefore, it is important to find a multi-target therapy for neuropathic pain.

Nitric oxide (NO) is involved in the modulation of peripheral and central nerve nociceptive pathways, especially in the spinal cord [4]. Regulation of NO is a delicate and complicated process, and NO synthase (NOS) is the rate-limiting enzyme for NO synthesis. NOS include three isoforms: nNOS, iNOS, and eNOS. It has been demonstrated that NOS inhibitors have protective effects against injury [5]. Thus, NOS may be involved in signal transduction in spinal cord nociceptive pathways and play a critical role in changes in synaptic plasticity and the pathophysiology of chronic pain [6]. Different NOS isoforms have distinct primary roles [7]. In neuropathic pain, the expressions and activities of the three NOS isoforms in the spinal dorsal horn remain unclear.

Hyperbaric oxygen (HBO) therapy is a commonly used medical treatment. HBO enhances the healing process by inhalation of a high concentration of oxygen in a total body hyperbaric oxygen chamber, where atmospheric pressure is over one atmosphere. HBO therapy is simple and easy to operate with minimal side effects. It is also reusable, has low medical cost, and is easily accepted by patients. HBO therapy has been widely used for a variety of neurological diseases, including acute spinal cord/peripheral nerve injury, focal cerebral ischemia, and even stem cell therapy. It has also been assessed for its effects on chronic pain [8]. It has been shown that HBO is effective in treating complex regional pain syndrome, multiple sclerosis, and migraines. More recent findings identified effects of HBO on several types of NP. Unfortunately, few studies have focused on the mechanism through which HBO therapy mediates NP. Studies have shown that HBO therapy can alleviate pain in two neuropathic pain models [9] and decrease tumor necrosis factor-alpha production [10]. In a traumatic brain injury model, HBO reduced oxygen-deprivation-induced brain damage and improved the ultrastructure of cortical neurons [11]. In a previous study, we found that a single HBO treatment [12] reduced mechanical and thermal sensitivity in rats and changed the expression of NOS isoforms in the spinal dorsal horn, but repeated hyperbaric oxygen therapy and the mechanism of action were not clear. Using hyperbaric oxygen therapy with different pressures and treatment time [13], our results showed that a short-term HBO treatment induced pain, which gradually diminished after 1 hour and then led to a long-term analgesic effect. HBO inhibited the activation of astrocytes and the production of inflammatory factors. P2X4R activation on the microglia and inhibition of caspase 3-mediated apoptosis also participated in the mechanism of analgesia [14]. These data indicated that the mechanism of HBO is complex, requiring further studies to elucidate the effect of repeated HBO treatments. Based on the results of these previous studies, we selected 2.5ATA for five consecutive days as the experimental treatment conditions.

In this study, rats were treated with HBO after a chronic construction injury (CCI) operation. The results of pain-related behavioral tests, ultrastructural changes, and NOS expression in the ipsilateral spinal dorsal horn were evaluated. These results provide a potential therapeutic target for neuropathic pain.

## RESULTS

### Observation of pain-related behaviors

Three days after the CCI operation, rats in the CCI group showed limping, left paw suspension, dorsiflexion with a continued paw contraction, stiffness of joints, and weight loss. However, rats in the HBO group demonstrated that their left hindlimbs were able to bear weight, with the palms placed on the surface, spread toes, and no limping observed.

**Figure 1:**
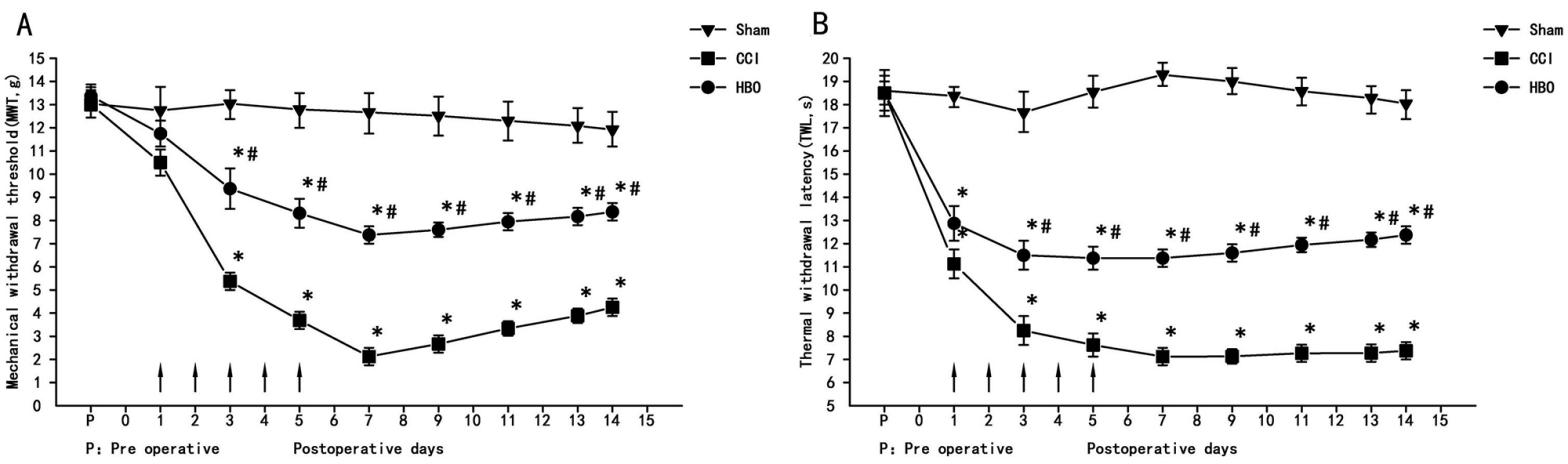
HBO treatment at early stages alleviates neuropathic pain Change in MWT and TWL in all rats at different days. **(A)** Changes in mechanical allodynia in all rats. **(B)** Changes in thermal hyperalgesia in all rats. HBO treatment was administered once daily for five consecutive days beginning from the first day after CCI, as indicated by arrows. Comparison of TWL and MWT in rats: ^*^P < 0.05 vs. sham; ^#^ P < 0.05 vs. CCI.

As shown in Figure 1, MWT and TWL were not changed in the sham group across all time points compared to a pre-surgery time point (P > 0.05). Rats in the CCI group showed a significant decrease in MWT 3 d after surgery (P < 0.05). The MWT of the CCI group reached its lowest threshold at 7 d after surgery, and this decrease was maintained as long as 14 d after surgery (Figure 1A). A similar change in TWL in the CCI group was observed (Figure 1B), but this measure reached plateau faster than the MWT. After 2.5 ATA HBO treatments for five days, rats from the HBO group showed a significant increase in both MWT and TWL (P < 0.05); suggesting early HBO treatment has significant effects on neuropathic pain.

### Electron microscopy

**Figure 2:**
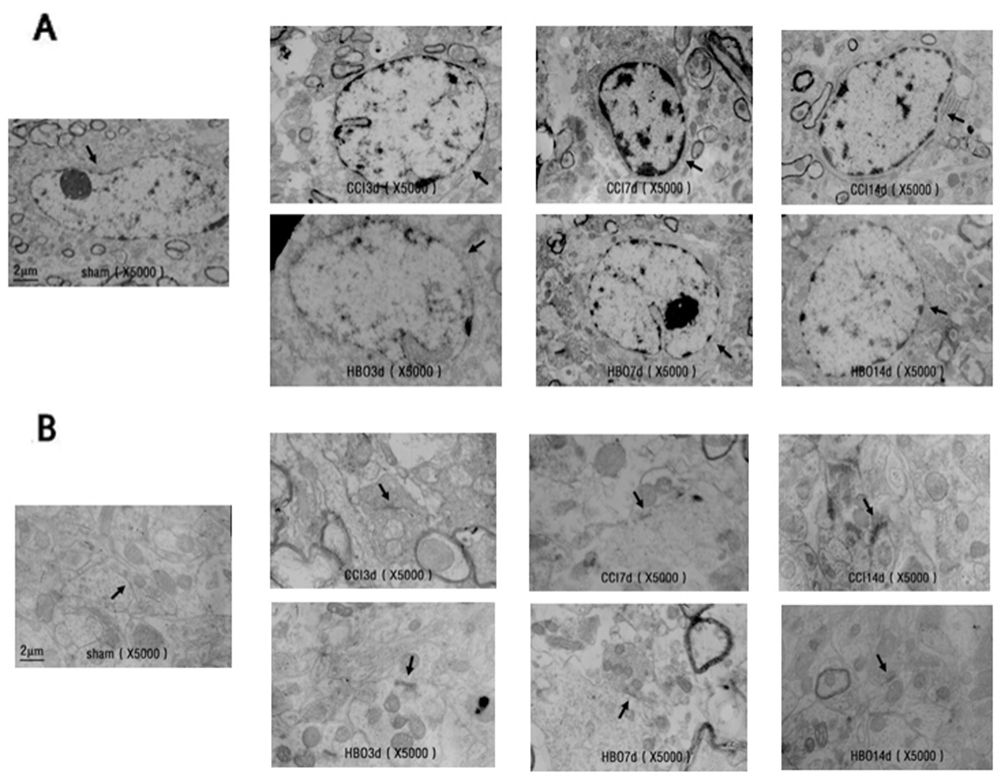
Electron micrographs showing the ultrastructure of spinal cord neurons and synapses in rats in the sham, CCI, and HBO groups at different days **(A)** spinal cord neurons; **(B)** spinal cord synapses. Magnification: 5000×.

As shown in Figure 2A, the nuclei of neurons in the sham group were round, with clear nuclear membranes and nucleolus, a uniform distribution of chromatin, and abundant cytoplasmic ribosomes (Ri), rough endoplasmic reticulum (REr), mitochondria (Mi), Golgi complexes (Go), lysosomes and other organelles. However, neurons from the CCI group showed near-round nuclei with damaged and unclear nuclear membranes, condensed chromatin, reduced Ri numbers, and a decrease in mitochondrial ridges with unclear mitochondrial outer membranes. Interestingly, these changes in the neurons observed in the CCI group were significantly improved in neurons from the HBO group (Figure 2A).

In the sham group, synaptic structures were clear with visible pre- and postsynaptic membranes and synaptic clefts. Synaptic vesicles were observed within presynaptic terminals, and the thickness of dense materials in the postsynaptic terminals was uniform. However, synaptic structures in the CCI group were disturbed, and synaptic membranes were fused between pre- and postsynapses. HBO treatment rescued CCI-induced membrane fusion and abnormal synapses. Synaptic structures in the HBO group demonstrated visible pre- and postsynaptic membranes and synaptic clefts, synaptic vesicles in presynaptic terminals, and uniform dense material in postsynaptic terminals, similar to those observed in the sham group (Figure 2B).

### Quantitative real-time PCR

**Figure 3:**
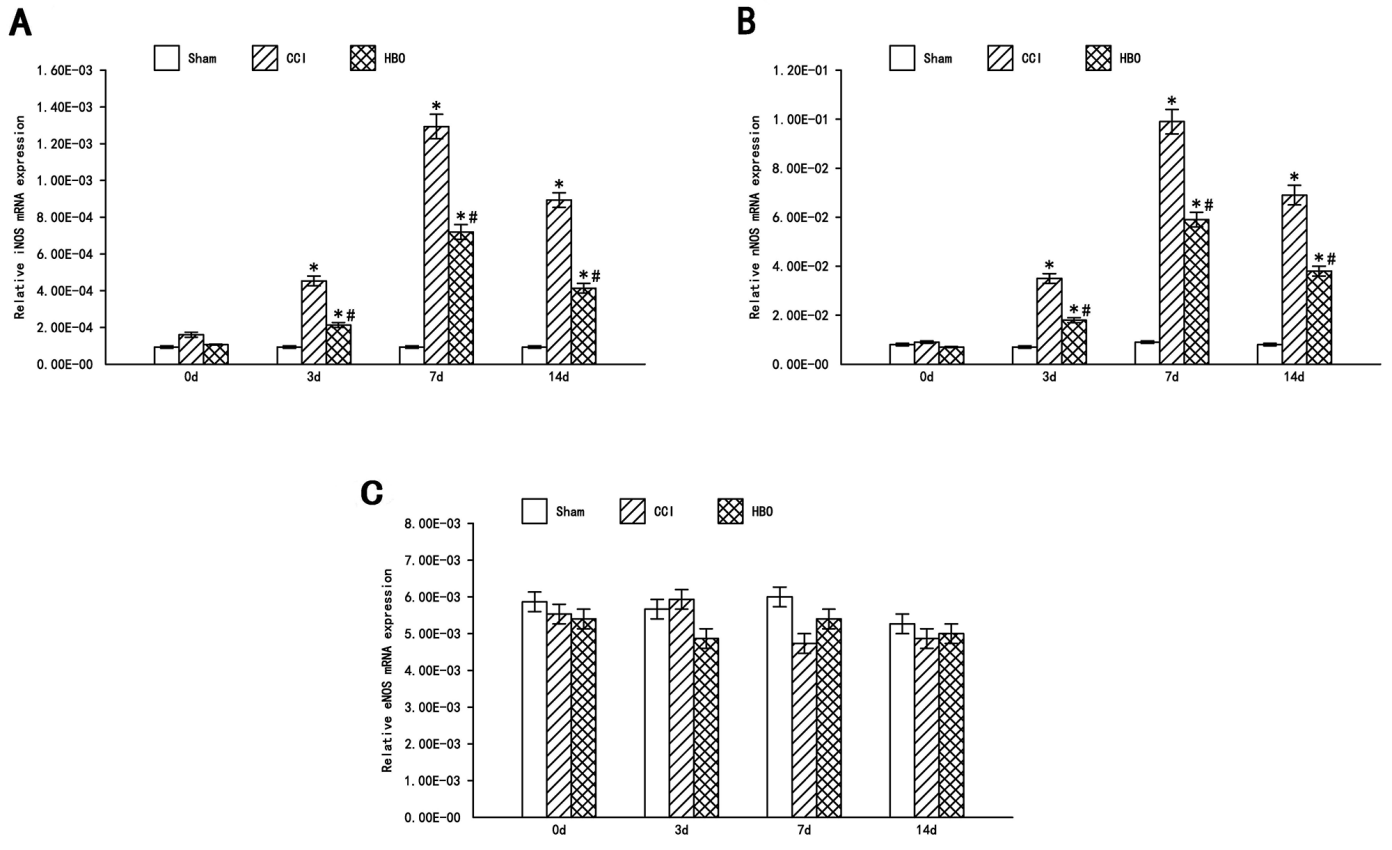
HBO attenuates the expression of iNOS and nNOS, but not eNOS in the spinal dorsal horn following CCI Real-time RT-PCR analysis of iNOS, nNOS and eNOS mRNA in the spinal dorsal horn. Statistical results: **(A)** Relative iNOS mRNA expression (iNOS/β-actin); **(B)** Relative nNOS mRNA expression (nNOS/β-actin); **(C)** Relative eNOS mRNA expression (eNOS/β-actin). Data are shown as mean ± S.E.M, n= 6. ^*^P<0.05 vs. sham; ^#^ P< 0.05 vs. CCI.

The relative mRNA expression levels of iNOS, nNOS, and eNOS in the ipsilateral spinal dorsal horn are shown in Figure 3. Compared to the sham group, the levels of iNOS in the CCI group were significantly increased 3 d after surgery (P<0.05), reaching a plateau at day 7 post-surgery; the levels of iNOS were decreased at day 14 but still expressed significantly (P<0.05) (Figure 3A). The levels of nNOS showed similar results (Figure 3B), while there was no obvious change in eNOS expression across the three groups (Figure 3C). iNOS and nNOS levels in the HBO group were significantly decreased compared to the CCI group (P<0.05), although they were still significantly increased compared to the sham group. Our results indicate that CCI does not induce eNOS expression, and that HBO treatment can decrease CCI-induced iNOS and nNOS expression, suggesting that HBO treatment has effects on neuropathic pain.

### Western blot analysis

**Figure 4:**
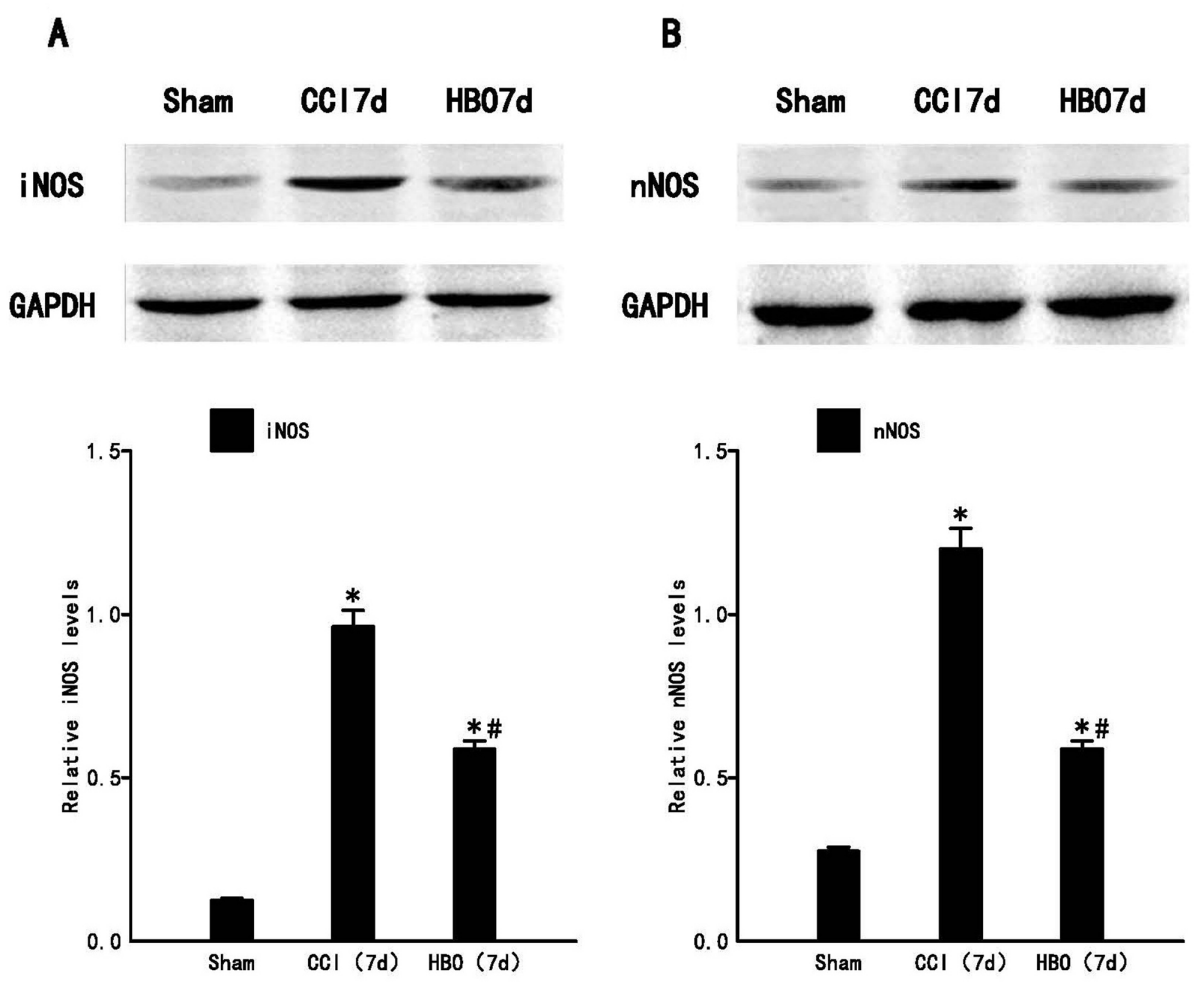
HBO treatment decreases the expression of iNOS and nNOS protein in the spinal dorsal horn significantly following CCI Western blot analysis of iNOS and nNOS on post-operative day 7. **(A)** The upper panel displays the bands of iNOS and GAPDH, and the lower panel indicates the statistical results of Western blot analysis. **(B)** The upper panel displays the bands of nNOS and GAPDH, and the lower panel indicates the statistical results of Western blot analysis. Data are shown as mean ± S.E.M, n= 6. ^*^P<0.05 vs. sham; ^#^ P< 0.05 vs. CCI.

Based on the real-time PCR data, the most significant time point (day 7) was selected. The protein levels of iNOS and nNOS in the ipsilateral spinal dorsal horn across the three groups were investigated. As shown in Figure 4, our results demonstrated that CCI significantly increases iNOS and nNOS protein levels compared to the sham group. HBO treatment significantly decreases the CCI-induced production of iNOS and nNOS proteins.

## DISCUSSION

Neuropathic pain is a common clinical disease, seriously affecting patients’ quality of life and ability to work. Significant investments have been made to find noninvasive, cost-effective treatments with few side effects.

In this study, MWT started to decline at 3 d after CCI operation, and reached the lowest value at 7 d after surgery. This decreased pain threshold was maintained as long as 14 d after surgery. TWL was also significantly shortened, with an earlier onset than the change in MWT. Consistent with previous studies, it indicates that our model is successful. These decreased thresholds indicate a possible association with inflammatory and neuropathic pain, with both peripheral and central nervous system sensitization. The CCI model has demonstrated similar symptoms as clinical neuropathic pain; therefore, it is widely used in neuropathic-pain-related studies [15]. After HBO treatment, the rats showed no limping, decreased spontaneous pain, increased MWT, and extended TWL. Thus, early continuous 2.5 ATA HBO treatments can effectively relieve hyperalgesia and neuropathic pain.

Neuropathic pain is closely related to anatomical reconstruction. Apoptosis and synaptic morphological abnormalities in spinal dorsal horn neurons are important factors in the development of neuropathic pain [16, 17]. Under physiological conditions, the peripheral receptors of primary sensory neurons exist in the spinal dorsal horn. Aβ fibers with low threshold exist in the spinal dorsal horn layers III and IV. C fibers with high threshold are present in layer II. Injuries to the peripheral nervous system can cause a reduction of inhibitory interneurons in spinal dorsal horn [18]. Neuronal apoptosis increases the spaces between synapses, and Aβ fibers can form abnormal “buds” and migrate into layer II, occupying the space normally associated with the axons of C fibers and creating new connections with nociceptive neurons [19]. Thus, these Aβ fibers can activate neurons that originally only responded to high threshold C fibers, eventually leading to hyperalgesia and allodynia. Electron microscopy results showed that the nuclei of neurons from the CCI group were nearly rounded, the cell membranes were partially damaged, the nuclear membranes were blurred or damaged, and the heterochromatin in the nuclei was condensed. Cytoplasmic ribosomes (Ri) were also decreased, the rough endoplasmic reticulum (REr) was expanded, mitochondrial (Mi) outer membranes were blurred, and synaptic structures were disturbed, with fused pre- and postsynaptic membranes. HBO therapy maintained the stability of cell membranes, reduced the damage to cell membranes, and alleviated local swelling of the nuclear membranes to reduce neuronal damage and abnormal synaptic formation. HBO therapy maintained sensory information transmission and integration through the dorsal horn neurons. Therefore, HBO may reduce pain through changes in the ultrastructure.

NO mediates various neurophysiological and pathological processes, such as neuronal survival and regeneration, regulation of synaptic plasticity, nerve injury, and neurodegenerative processes. NO induces neuronal apoptosis in inflammatory pain [20]. NOS is the rate limiting enzyme for NO synthesis. Studies have shown that NOS plays an important role in inflammatory pain. However, in neuropathic pain, the role of NO is debated. On the one hand, it is thought that in neuropathic pain, afferent neurons in the spinal cord release glutamate to activate NMDA receptors, causing Ca2+ influx and the activation of NOS. The NO synthesized by NOS disperses to the presynaptic membrane via the cell membrane and further promotes glutamate release and enhances pain [21]. On the other hand, several studies have indicated that NO and cGMP can activate target molecules, including PKG and different types of potassium channels, resulting in analgesic effects. The activation of the NO-cGMP-PKG signaling pathway can potentiate ATP-dependent potassium channels and result in protective effects against injury [22-24]. In the present study, we examined the mRNA levels of different NOS isoforms. We found that the expression levels of iNOS and nNOS in CCI group were significantly higher than the sham group, and reached a peak at day 7 after surgery. This trend correlated with the decline in MWT and TWL. No significant changes were observed in eNOS mRNA levels. Thus, hyperalgesia in rats was associated with the increase in iNOS and nNOS, which play a major role. Arnau Hervera et al found [25] that iNOS-regulated nNOS increase participated in the maintenance of neuropathic pain in mice, with no eNOS increased in spinal, consistent with our results.

Activation of the NO-cGMP-PKG-KATP signaling pathway [26] is involved in the regulation of pain in both the peripheral and central nervous systems. In the peripheral nervous system, this pathway activates the anti- analgesia effects of morphine; while in the central nervous system, it increases μ-opioid receptor (MOR) expression in the dorsal root ganglions (DRGs). HBO treatment can improve blood oxygen concentration, improve local microcirculation, and accelerate the clearance of reactive oxygen species (ROS). iNOS and nNOS expression in the spinal dorsal horn were significantly reduced after HBO treatment, and this effect was sustained to 14 d. The early analgesic effects of HBO may involve the reduced production of tumor necrosis factor-alpha [10], but long-term analgesic effects may be associated with the induction of NO-dependent opioid peptide release [27]. HBO can decrease the expression of iNOS and nNOS in both the protein and gene levels, and provide protective effects against neuronal injury by regulating spinal NOS isoforms/NO content [28-30]. Therefore, we will further investigate the mechanisms of iNOS and nNOS in regulating neuropathic pain.

In conclusion, early 2.5 ATA HBO can effectively relieve CCI-induced neuropathic pain in rats, maintain neuronal structures in the spinal dorsal horn, reduce abnormal synapse formation, decrease the expression levels of iNOS and nNOS in the spinal cord, and alleviate pain. Therefore, changing the expression levels and isoform ratios of iNOS and nNOS is one of the mechanisms of HBO treatment in reducing neuropathic pain. Early HBO relieves mechanical allodynia and thermal hyperalgesia via activation of the NO-cGMP-PKG signaling pathway [31].

## MATERIALS AND METHODS

### Animals

This study was carried out in accordance with the Guidelines for the Care and Use of Laboratory Animals of the National Institutes of Health and the Animal Welfare Act. All protocols were approved by the animal care committee at Shengjing Hospital of China Medical University (2016PS063K). Animals were purchased from the Experimental Animal Facility at Shengjing Hospital of China Medical University. The experiments used male Sprague-Dawley rats (8–10 weeks old, weighing 270–290 g), and there were no significant differences among the three groups’ biological data. All rats were single housed in a temperature-controlled (23–25°C) room with natural light and relative humidity of 40%–60%. Food and water were provided freely. All animals were randomly assigned to the sham, CCI, or HBO group. After surgery, the CCI group and HBO groups were further divided into four sub-groups: 0 d, 3 d, 7 d and 14 d post-surgery.

### Chronic constriction injury model

The CCI model utilized in this study was performed according to Bennett and Xie [32]. In brief, all animals were anaesthetized with chloral hydrate (intraperitoneal injection, 300 mg/kg). A blunt dissection through the biceps femoris was made at the mid- thigh level on the left leg, and the sciatic nerve was exposed. Four silk ligatures (4.0 chromic gut) were tied loosely around the sciatic nerve with an interval distance of 1 mm approximately. Ligatures were tied with great care such that the nerve was just barely constricted, producing a brief twitch in the muscle surrounding the sciatic nerve. Wounds were closed by layered suturing and animal were returned to their home cages for recovery. Compared with baseline values, animals with MWT and TWL reductions >20 % on the first postoperative day were included in subsequent experiments. Animals in the sham group received the exact same procedures without ligation of the sciatic nerve.

### Hyperbaric oxygen therapy

Prior to HBO treatment, fresh lime was placed in the bottom of the HBO chamber (DS400-IV, Weifang Huaxin Oxygen Industry Co., Ltd., Shandong, China) to reduce the accumulation of H2O and CO2. The HBO chamber was washed with pure oxygen for 10 min to ensure the oxygen concentration was >90%. Animals in the HBO group then were placed in the chamber. The pressure in the chamber was gradually increased at a rate of 0.125 ATA/min until reaching 2.5 ATA, and maintained at 2.5 ATA for 60 min. After treatment, the pressure was resurfaced to atmospheric pressure within 20 min at a constant rate. Animals in the HBO group received treatment one day after CCI operation for five consecutive days. During HBO treatment, animal behaviors were closely observed. Animals in the sham and CCI groups were simply placed in the chamber for the same duration, but did not receive any treatment.

### Observation of pain-related behaviors

All animals were single housed and gait, left hindlimb posture, with or without autophagy, bearing burden of the hindlimb, and body weight were observed after the operation. Pain-related behaviors were also assessed at various time points, as follows:

### Spontaneous paw withdrawal

Rats were placed in a glass chamber and allowed free movement for 5 min. The number of spontaneous left hind paw withdrawals was recorded.

### Mechanical withdrawal threshold (MWT) test

The MWT was determined for each hind paw utilizing von Frey filaments (Stoelting, Chicago, USA) ranging from 0.14 g to 15 g and an “up and down” procedure from 10 am to 12 pm every day post-surgery. In brief, rats were placed in a Plexiglas chamber on top of a raised platform that allowed easy administration of the mechanical stimuli. Animals were allowed to acclimatize for 15 min prior to each test session. A series of von Frey hairs was applied from below the customized platform to the plantar surface of the hind paw in ascending order beginning with the lowest hair (0.14 g). A particular hair was applied until buckling of the hair occurred and it was maintained for approximately 3 s. The applications were spaced in 15-s intervals. If a withdrawal response to a particular hair was observed at least five times, the value of that hair in grams was considered to be the withdrawal threshold. If a withdrawal response did not occur with the 15 g von Frey filament, it was considered a painless response. A paw withdrawal due to animal movement was not considered a positive response.

### Thermal withdrawal latency (TWL) test

TWL was assessed to determine the rats’ thermal sensitivity. Rats were placed on the surface of a thermal testing apparatus and allowed to acclimatize for 15 min prior to each test session. A radiant heat source (Youer Equipment Scientific Co., Ltd., Shanghai, China) located under the glass was focused onto the hind paw of each rat. The paw TWL was recorded by a timer three times with 10-min intervals between each trial, and the mean of these three trials was determined. A cut-off time of 30 s was used to prevent potential tissue damage. If no paw withdrawal occurred by 30 s, the radiant heat was stopped and TWL was recorded as 30 s.

### Sample preparation

At 0, 3, 7 and 14 d after the surgical operation and pain-related behavioral tests, six rats from each group were anesthetized by intraperitoneal injection of chloral hydrate solution (300 mg/kg), followed by a cervical dislocation. The L4–L6 spinal cord segments were removed on ice and the ipsilateral spinal dorsal horns were dissected immediately and kept in liquid nitrogen until use.

### Electron microscopy

Spinal dorsal horns were fixed using 2.5% glutaraldehyde and cut into 0.2 cm × 0.2 cm blocks for electron microscopy. Tissues blocks then were washed with phosphate-buffered saline (PBS) and post-fixed in 1% osmium tetroxide, followed by dehydration in a series of acetone washes and resin embedding in EPON812. Samples were sectioned into 60–70 nm and double stained with uranyl acetate and lead citrate. Ultrastructure was observed and images were obtained by electron microscopy.

### Real-time PCR

Samples were lysed in Trizol solution (Invitrogen, Carlsbad, CA, USA) and total RNA was extracted according to the manufacturer’s protocol. RNA concentration was measured using a spectrophotomer, and cDNA was synthesized by reverse transcription. The primer sequences of iNOS were as follows: upstream primer 5’-ATC CCG AAA CGC TAC ACT T-3’; downstream primer 5’-CGG CTG GAC TTC TCA CTC-3’. The primer sequences of nNOS were as follows: upstream primer 5’-GGG GCT CAA ATG GTA TGG-3’; downstream primer 5’-TCT TCT TGG CTA CTT CCT CC-3’. The primer sequences of eNOS were as follows: upstream primer 5’-CAC AGG CAT CAC CAG GAA-3’; downstream primer 5’-CAG AGC CAT ACA GGA TAG TCG-3’. The primer sequences of β-actin were as follows: upstream primer 5’-TGG CAC CCA GCA CAA TGA A-3’; down-stream primer 5’-CTA AGT CAT AGT CCG CCT AGA AGCA-3’. Real-time PCR of NOS isoforms and β-actin was performed in Thermal Cycler Real-Time PCR machine (Takara, Kyoto, JPN) using the following program: 95°C for 30 s, 95°C for 5 s, 60°C for 30 s, and 72°C for 10 s. A total of 45 cycles were run followed by a melting curve analysis.

### Western blot analysis

Samples were homogenized in lysis buffer on ice, then centrifuged at 12,000 rpm for 10 min (4°C). Supernatants were collected and protein concentration was determined using a BCA assay. Proteins (5 μg) were separated by 10% SDS-PAGE under reducing conditions and electrophoretically transferred to a polyvinylidene difluoride (PVDF) membrane (Amersham Bio-sciences, Freiburg, Germany). Membranes were blocked in a TBST (Tris-buffer saline containing 0.05% Tween-20) solution containing 5% nonfat milk and then probed (4°C; overnight) with an anti-nNOS antibody (1:500, Epitomics, Burlingame, CA, USA), an anti-iNOS antibody (1:500, Millipore Corp, billerica, MA, USA), or an anti-GAPDH antibody (1:1000, Sigma Chemical Co, St. Louis, MO, USA), diluted in TBST containing 5% nonfat milk. Following washes in TBST buffer, horseradish peroxidase-conjugated secondary antibody (1:2000, ZSGB-BIO, ORIGENE, Beijing, China) was used to detect primary antibodies (2 h at room temperature). ECL plus reagent was used to develop films and the intensity of bands was determined by Gel-Pol analyzer.

### Statistical analysis

Data analysis was performed using SPSS version 16.0 software. Metrological data were first tested for normality. Data exhibiting a normal distribution were expressed as the mean ± standard deviation; Data exhibiting nonparametric distribution were expressed as the median and quarterback spacing. Behavioral tests were analyzed by a repeated measures analysis of variance (ANOVA). The data were first subjected to a Mauchly’s test of sphericity test. Our data satisfied the test of Mauchly’s test of sphericity condition. The two pairs of comparisons between the three groups at each time point were calculated by the multivariate analysis of variance in the general linear model followed by the Scheffe post hoc test. Real-time PCR and western blot data were analyzed by a one-way ANOVA followed by the Scheffe test for multiple comparisons. Differences were considered to be statistically significant at P < 0.05.
